# Epidemiology of Unintentional Child Injuries in the Makwanpur District of Nepal: A Household Survey

**DOI:** 10.3390/ijerph121214967

**Published:** 2015-11-30

**Authors:** Puspa Raj Pant, Elizabeth Towner, Matthew Ellis, Dharma Manandhar, Paul Pilkington, Julie Mytton

**Affiliations:** 1 Centre for Child and Adolescent Health, University of the West of England, Bristol BS8 2BN, UK; towner146@btinternet.com (E.T.); julie.mytton@uwe.ac.uk (J.M.); 2 School of Social and Community Medicine, University of Bristol BS8 2BN, UK; m.ellis@bristol.ac.uk; 3 Mother and Infant Research Activities (MIRA), Kathmandu 44600, Nepal; dsm@mira.org.np; 4 Faculty of Health and Applied Sciences, University of the West of England, Bristol BS16 1QY, UK; paul.pilkington@uwe.ac.uk

**Keywords:** child injury, unintentional injuries, community-based, household survey, Nepal

## Abstract

Secondary sources of information indicate that the proportion of child deaths due to injuries is increasing in Nepal. This study aimed to describe the epidemiology of unintentional injuries in children, explore risk factors and estimate the burden faced by families and the community in the Makwanpur district. We conducted a household survey in Makwanpur, covering 3441 households. Injuries that occurred during the 12 months before the survey and required treatment or caused the child to be unable to take part in usual activities for three or more days were included. We identified 193 cases of non-fatal unintentional child injuries from 181 households and estimated an annual rate of non-fatal injuries of 24.6/1000 children; rates for boys were double (32.7/1000) that for girls (16.8/1000). The rates were higher among the children of age groups 1–4 years and 5–9 years. Falls were the most common cause of non-fatal child injuries followed by burns in preschool children and road traffic injuries were the most likely cause in adolescence. Mean period of disability following injury was 25 days. The rates and the mechanisms of injury vary by age and gender. Falls and burns are currently the most common mechanisms of injury amongst young children around rural homes.

## 1. Introduction

The World Report on Child Injury Prevention concluded that low- and middle- income countries bear the biggest burden of child injuries [1]. Globally about one million children die from preventable injuries every year [1,2,3]. The South-East Asia Region alone had more than one-third of injury related deaths in the world in 2008 [1,4], but many South-East Asian countries do not have evidence from robust community-based surveys [5]. Injury experts have recommended the use of such surveys for increasing the knowledge-base on child injuries in low- and middle-income countries [1,6,7,8].

Nepal is among the 49 least developed countries (LDCs) in the world with 39% of the population living below the poverty line of US $1.25/day [9]. Child mortality rates are still very high, despite having achieved a two-thirds reduction in the past 20 years [10]. In the year 2010, about 37,000 children below five years of age died in Nepal, including 55% of deaths which occurred within four weeks of birth. The Global Burden of Disease Study estimated that in 2010 injuries were responsible for about 18% of total deaths among Nepalese children aged 1–14 years [3]. This study also estimated that injuries were responsible for 11% of total DALYs and 18% of YLLs for the children of this age group [3]. The fatal injury rate for children was as high as 70 per 100,000 for children aged 5–9 years, as estimated by GBD 2010 [3].

Fatal injuries are considered ‘the tip of the iceberg’ of a range of injuries [6,11]. Rates of non-fatal injuries are typically many times higher than fatal injuries. Such injuries to children may have long term consequences proportionally greater than adult injury due to the remaining years of life affected by disability [12]. Because of deep-rooted fatalistic perceptions about injuries [13], challenging environmental factors and lack of awareness, many non-fatal injuries in Nepal are ignored. This study is the first attempt to explore the epidemiology of severe non-fatal child injuries in Nepal using a community-based survey approach [5]. This paper identifies the types of child injuries, their risk factors and estimates the burden faced by families. This information will help to design appropriate injury prevention interventions in similar settings. 

## 2. Methods and Materials 

This study used a cross-sectional survey design and was conducted in the Makwanpur district in the Central Region of Nepal. The district has a population of 420,000 (45% are children below 18 years of age) living in 86,000 households in 43 village development committees (VDCs) and one municipality [14]. Makwanpur district’s Human Development Index ranks 31st out of the total of 75 districts in Nepal [15]. The district centre is Hetauda, where 20% of the district’s total population live [15]. The study team worked with Mother and Infant Research Activities (MIRA, www.mira.org.np), an established Non-Governmental Organisation (NGO) based in Makwanpur district. This NGO has experience in conducting detailed household mapping and community health surveys in this area [16]. Makwanpur district has a mix of geographical environments ranging from low-land (<500 m) to high hills (up to 3500 m) [17] and it encompasses a range of socio-economic settings found in Nepal [16]. Results from this survey thus have the potential to be generalizable for many districts in Nepal.

### 2.1. Sampling

A sample size of 3520 households was calculated, based on UN guidelines for household surveys [18]. This took into account an estimate of injury incidence of 5.5% based on the literature from Asian countries [19]; a non-response rate of 10%; average household size of 5.5 persons; a proportion of child population of 45%; and a precision of 0.006 (10% of the estimate). A household was defined as a group of individuals living together and sharing the same kitchen. MIRA had previously conducted a cluster randomised trial on neonatal health; for which 21 pairs of VDCs were matched on the basis of population; geography and ethnicity [16,20]. Twelve out of those 21 pairs were randomly selected and one VDC per pair was randomly allocated to receive the intervention and other to act as a control [16,20]. The 12 VDCs included in this survey were those from the original control group of the MIRA study. The only municipality, Hetauda, was added to capture information on urban settings.

**Figure 1:**
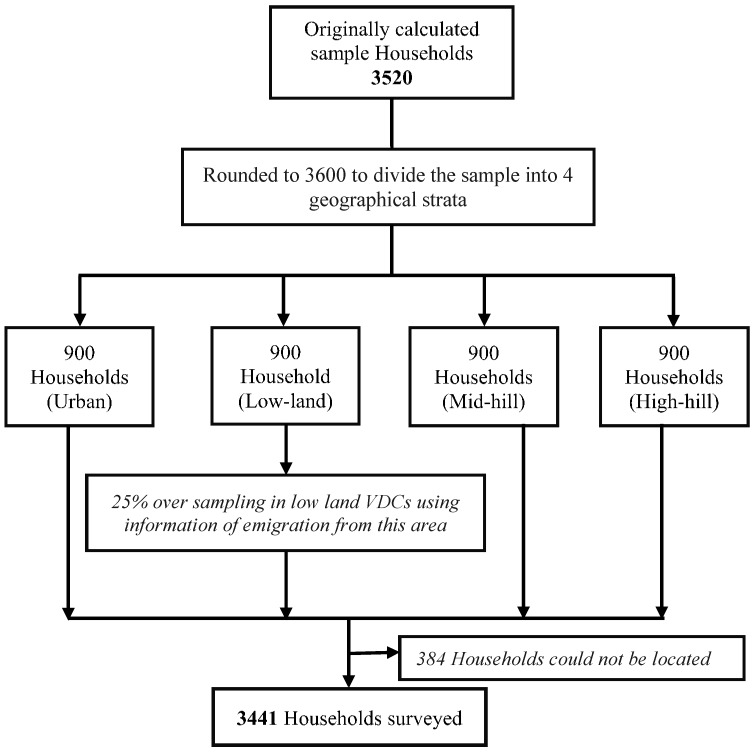
Flowchart of the sampling and household selection.

It was hypothesised that there would be differences in the lifestyles and risk factors for injuries according to geographical settings resulting in variations in the injury types. The area was divided into four geographical strata of high hills, mid hills, low land and urban area according to their elevation and location, with 900 households sampled in each stratum (Figure 1). Microsoft-Excel generated random numbers were used to determine the survey household using an existing list of households held by MIRA (for VDCs) and on the electoral roll for Hetauda.

### 2.2. Definitions

An injury case was defined as damage to body/organs due to an unintended external force that may have required treatment, or resulted in missing school or work or being unable to carry out normal activities for at least three days [21]. It included fall related injuries, injuries caused by sharp objects, drowning, poisoning, transport injuries, electric shocks, burns/fire, suffocation, injuries caused by falling objects, injuries caused by machines and injuries caused by animals. This study excluded intentional injuries such as suicide or suicidal attempts, homicide and violent assaults or injuries related to war/crime. A child was defined as a person less than 18 years of age [1].

### 2.3. Data Collection

The survey collected information on households’ demographic and socio-economic variables such as household assets and facilities, cooking fuel, drinking water source and monthly household expenditure, as well as swimming and cycling ability to assess the skills of the children that may be associated with injury risk.

The face-to-face survey was conducted by local interviewers in sample households using structured questionnaires, adapted from the WHO Guidelines [22] and translated into the Nepali language. A pilot survey of 40 households in similar settings to the survey VDCs was carried out to test the questionnaires. A two-day orientation training for interviewers was conducted before the pilot survey and a review meeting was conducted afterwards; modifications to the questionnaires were then made. In total, four questionnaires were developed and used in the survey: (i) screening; (ii) injury morbidity; (iii) injury mortality and (iv) burden of injury questionnaires (copies of the survey tools are available from the corresponding author upon request). Screening questionnaires were used to identify households with injured persons; information on demographic and socio-economic variables were collected for all family members from each of the sample households. The households were also asked if any child had died from an injury during the previous 5 years, if so the injury mortality form was completed.

Questionnaires were completed through an interview with the head of the household or a responsible adult in the household. Children over 12 years were considered eligible for interview, if no other adult was available. Injuries were recorded if they occurred in the 12 month period between 16 December 2009 to 15 December 2010; corresponding to the first day of 9th month of the Year 2066 to the last day of 8th month of 2067 of the Nepali calendar. The commencement of this study also corresponded to the end of Diwali festivals which also helped participants remember the milestone. A 12 month recall period was applied to capture seasonal variations in injuries. In order to improve the recall of the respondents, screening questions about past illnesses were asked for all household members to include: (i) any illness in the past year; (ii) any injury in the past year and (iii) whether the injury was of unintentional type. A list of the types of mechanisms of injuries was available for the interviewer to help respondents correctly categorise events. Each unintentional injury case reported was recorded on a separate data collection form.

The interviewers, employed with MIRA, were also residents of the surveyed VDC areas. Therefore they found it straightforward to locate the survey household because of their knowledge of the area and their previous experience working on local surveillance projects. Two VDCs affected by flooding and landslides were reported to have experienced recent significant outward migration; a precautionary oversampling of 25% was applied in these VDCs (Figure 1).

Potential biases were anticipated and attempts were made to address them beforehand by: (i) developing screening questions; (ii) defining injuries and reading out the definition of injury to all respondents; (iii) having a fixed recall period highlighted by festival dates; (iv) mobilising local interviewers; and (v) conducting interviews in front of family members. Interviewers were provided with a two-day orientation training aided by a detailed Interviewer’s manual (prepared by the lead author in Nepali language). An operational definition of injury was read out by the interviewers to the respondents to explain what was meant by injuries and what types of injuries were included. Ethical approval for conducting the study was obtained from the Nepal Health Research Council. Verbal consent was obtained from all the participants before the interview.

### 2.4. Data Analysis

Data were entered into an MS-Access database and were analysed using SPSS 18.0 [23]. Rates and proportions for different types of injuries were calculated and reported by demographic variables. The injury incidence rates with 95% confidence intervals were calculated by age groups, gender, area of residence and types of injuries/mechanisms. 95% Confidence Intervals were calculated using Epi-info web application (http://wwwn.cdc.gov/epiinfo/). Significance of non-parametric analyses using chi-square tests were estimated using *p* values.

## 3. Results 

### 3.1. Survey Population

Screening information was collected for 20,346 individuals of all ages living in 3441 households in Makwanpur district. Within this sample there were 7844 (39%) children of less than 18 years of age, living in 2868 households. Overall, the survey achieved a response rate of 97.8% eligible households. No household declined to participate in the survey, however, 2% of the sample households could not be located as they might have moved permanently.

A majority (59%) of the survey households consisted of between five and eight people. Only one-fifth of the people had regular sources of income such as a salaried post or waged labour work (either in Nepal or abroad). 31% of the survey population lived on subsistence agriculture. The remaining half of the population self-reported as students (34%), unemployed (9%) and housewives (7%). One in five people reported “any illnesses” during the last year amongst which injury was identified by one in five.

### 3.2. Incidence of Non-Fatal Injuries

A total of 193 (2.5%) children with non-fatal injuries were identified in the previous 12 months. The key indicators and incidence rates are presented in Table 1. The overall incidence rate for boys was 32.5/1000, nearly double that of the girls (16.8/1000). The non-fatal injury rates (per 1000) were the highest among the 5–9 year age group and lowest among the 15–17 year age group. The injury incidence rate in rural areas was slightly lower than in urban areas; very low rates were reported in high-hill areas. There was no significant difference in injury rates for different occupational groups of children. This study did not find any injured infant in the sample population.

**Table 1:** Annual incidence of non-fatal injuries in Makwanpur district, Nepal.

	Surveyed Children (*n =* 7844)	Children Identified with any Non-Fatal Injury	Annual Incidence of Non-Fatal Injury per 1000 Children (95% CI)	*p*
*Gender*				
Male	3906	127	32.5 (27.4–38.6)	<0.001
Female	3938	66	16.8(13.2–21.3)	
*Age Group*				
1–4 years	1276	37	29.0 (21.1–39.7)	0.002
5–9 years	2044	62	30.3 (23.7–38.7)	
10–14 years	2651	69	26.0 (20.6–32.8)	
15–17 years	1601	25	15.6 (10.6–23.0)	
*Occupation*				
Minor (<10 years)	1694	40	23.6 (17.4–32.0)	0.914
Agriculture	576	14	24.3 (14.5–40.4)	
Students	5245	131	25.0 (21.1–29.6)	
Labourer	163	4	24.5 (9.6–61.4)	
Others	166	4	24.1 (9.4–60.3)	
*Geographical Region*				
High Hill	1786	26	14.6 (10.0–21.2)	0.01
Mid Hill	2327	64	27.5 (21.6–35.0)	
Low-Land	2479	71	28.6 (22.8–36.0)	
Rural (Overall)	6592	161	24.4 (21.0–28.4)	
Hetauda (Urban)	1252	32	25.6 (18.2–35.9)	
OVERALL	7844	193	24.6 (21.4–28.3)	

The rates of non-fatal injury were higher among males of all age groups were found to be highest in the two youngest categories. In contrast for girls, rates of injury were similar across the lowest three age categories (Table 2).

**Table 2:** Rates (per 1000) of non-fatal injuries by gender and age groups.

Age Group	Male (Rate (95% CI))	Female (Rate (95% CI))
1–4 years	38.8 (26.2–57.0)	19.8 (11.6–33.6)
5–9 years	40.9 (30.6–54.4)	18.6 (11.8–29.2)
10–14 years	32.3 (24.0–43.4)	20.0 (13.8–28.9)
15–17 years	21.9 (13.7–34.9)	9.7 (4.9–19.0)
Over all	32.5 (27.4–38.6)	16.8 (13.2–21.3)

Pearson-Chi Square *p* values for age = 0.671; df = 3.

### 3.3. Type of Injuries

Falls were the most frequent category of non-fatal injury reported in all age groups (Table 3) and were most commonly occurring from trees, balconies, ladders or edges of fields.

**Table 3:** Rates (per 1000) of non-fatal injuries by age groups, gender and mechanisms.

Mechanism of Injury	Age *				Gender **		Total
	1–4 Year (95%CI)	5–9 Year (95%CI)	10–14 Year (95%CI)	15–17 Year (95%CI)	Male (95%CI)	Female (95%CI)	Rate (95%CI)
Falls	11.0	19.6	12.4	4.4	15.9	8.1	12.0
	(6.5–18.3)	(14.4–26.5)	(8.9–17.4)	(2.1–9.0)	(12.4–20.3)	(5.8–11.5)	(9.8–14.6)
Burns/Scalds	8.6	2.4	0	0.6	3.1	1.3	2.2
	(4.8–15.4)	(1.0–5.7)	(0)	(0.1–3.5)	(1.8–5.4)	(0.5–3.0)	(1.4–3.5)
Cut/Crush	6.3	3.4	4.1	3.1	4.1	3.8	4.0
	(3.2–12.3)	(1.7–7.1)	(2.3–7.4)	(1.3–7.3)	(2.5–6.6)	(2.3–6.3)	(2.8–5.6)
Traffic Related	1.6	2.0	3.8	3.7	4.6	1.0	2.8
	(0.4–5.7)	(0.8–5.0)	(2.1–6.9)	(1.7–8.2)	(2.9–7.2)	(0.4–2.6)	(1.9–4.2)
Hit by Object	0.8	10.5	1.5	1.2	1.5	0.5	1.0
	(0.1–4.4)	(0.1–2.8)	(0.6–3.9)	(0.3–4.5)	(0.7–3.3)	(0.1–1.9)	(0.5–2.0)
Other Injuries	0.8	2.4	4.1	2.5	3.3	2.0	2.7
	(0.1–4.4)	(1.0–5.7)	(2.3–7.4)	(1.0–6.4)	(1.9–5.7)	(1.0–4.0)	(1.8–4.1)

Pearson-Chi Square *p* values for * age < 0.001; df = 15 and ** gender = 0.316; df = 5.

There was no discernible pattern to the children’s activities at the time of fall injuries. The second leading mechanism of nonfatal injuries was different for different age groups. Burns were the second leading cause of injury for children aged 1–4 years, and being hit by moving objects for children aged 5–9 years. Cut/crush injuries and road traffic injuries (RTIs) were the second leading cause of injuries among children aged 10–14 years and 15–17 years, respectively (Table 3). RTIs and cut/crush injuries were the second leading cause of injuries among boys and girls, respectively.

### 3.4. Place of Injuries

As shown in Table 4, overall 39% of children were injured in the home environment; for children aged 1–4 years this amount was over 80%. The proportion of injuries occurring at home declined with increasing age. The proportion of injuries occurring in the road/street was 19%. In rural Nepal, children also accompany adults to the fields or other work places, resulting in 17% of injured children of all ages reported to be injured at farms and/or animal sheds.

It may be expected that a greater number of children will get injuries at home, in the road/street or at school but the proportion of injuries occurring to children in the jungle, in farms/animal sheds and at markets/construction areas was not anticipated. This finding suggests that many children may have access to hazardous places without supervision, possibly due to a lack of understanding of the risk of injuries. The circumstances around these children’s injuries included examples of collecting fuel/wood or fodder, shopping for daily household supplies, and supporting parents in agricultural work.

**Table 4:** Distribution of injured children by places of injury.

Places of Injury	Injured Children (%)	Rate/1000 Children
Home	75 (38.9)	9.6
Road/Streets	37 (19.2)	4.7
Farm/Fields/Animal Sheds	33 (17.1)	4.2
Jungle	29 (15.0)	3.7
School	15 (7.8)	1.9
Market/Construction Sites	4 (2.1)	0.5
Total	193 (100.0)	24.6

## 4. Discussion 

To our knowledge these are the first population-based data on non-fatal child injuries in Nepal, collected by a systematic household survey. An overall rate of incidence of unintentional non-fatal injury was found to be 24.6 per 1000 children (95% CI 21.4–28.3). When extrapolating this incidence for the entire district, an estimated 3987 (95% CI 3874–4117) children aged 0–17 years may have non-fatal injuries annually in Makwanpur district alone. These figures highlight a considerable burden of moderate or severe injuries in the population of Makwanpur.

The injury incidence rate observed in Makwanpur is higher than that of 15.9 per 1000 reported from a study in Bangladesh [23] and that of 15.4 per 1000 reported for a study in Thailand [24]. Although, our study uses similar definitions of injury, there was one difference in that we included injuries regardless of treatment status. In the Bangladesh and Thailand studies the injuries were counted only where medical treatment was sought whereas in our study we counted injuries regardless of medical treatment as treatment options are relatively limited in Nepal. Falls were the mechanism of injuries for over half of all injured children (51%); the majority of these falls occurred whilst the child was playing, helping parents in agricultural work or during school journeys. About 5% of non-fatally injured children received injuries from thorns, nails, or other pointed objects resulting in skin punctures. According to general observation in the field, this may be because children do not have protective footwear to wear. These are the most neglected types of injuries in rural areas. These findings have implications for injury prevention in home, school and travel settings.

The variable magnitude of the problem found in the literature is linked with the techniques and definitions of injury applied in the study; a systematic study of community-based studies in South-East Asian countries found contrasting results [5]. Studies from Pakistan described higher rates of injury (46 per 1000) for pre-school children, compared with our findings. The Pakistan study used a different methodology: an interview followed by individual health examination [25,26]. Therefore, direct comparison of these results is not possible. Our study found falls as the most common type of non-fatal injury; fall injuries were the leading cause of non-fatal injuries in most of the community-based studies conducted in Vietnam, the Philippines and China, while RTIs were the leading causes in Thailand [19,24]. This study used the same definition used in the surveys from Bangladesh, Philippines and Thailand *i.e.*, not being able to carry out usual activities for 3 days while the studies from China and Vietnam used a time period of 1 day or presentation to a professional [27].

In our study non-fatal injury rates were found to increase with age in children aged 1 to 17 years. This was similar to a study conducted in Macedonia [28] which found child injury rates ranging from 34/1000 for children aged 5–9 years to 103/1000 for adolescents aged 15–19 years. This study included both minor and major injuries using 6 and 12 months recall periods respectively. However, a survey from Sri Lanka [29] reported a decreasing trend in non-fatal injury rates with age this may be because of higher proportion of children going to school in Sri Lanka, which is a relatively well supervised environment.

There are a few community-based studies from Nepal that report child injuries [5]. The annual incidence rates for non-fatal child injuries (0–9 years) was found to be as high as 545 per 1000 children in Dharan Municipality [30] and a school-based survey of children aged 10–17 years [31] found the non-fatal injury rates ranging from 225 to 650 per 1000. Both these studies were cross-sectional and were conducted in urban settings only. These estimates are not suitable for direct comparison with those found in this study because they included minor lacerations and wounds and employed a short recall period of one month [5]. A hospital-based study of 11 hospitals in Nepal found that children below 15 years of age comprise 25% of all the hospital visits due to injury [32]. Collectively these data indicate that child injuries pose a significant burden on Nepal’s public health. Rapidly changing developmental stages during childhood and their changing interaction with surrounding environments results in varying levels of injury morbidity and mortality. Knowledge about injuries by age are useful to address the problem accordingly [1].

All but one of the injuries reported in our survey were collected from someone other than the injured child, *i.e.*, one adult household member responding for all of the children in the household. However, we aimed to speak to the victim where this was possible and if s/he was over 12 years of age. Using a proxy source for interview may result in an underreporting of non-fatal injuries [33,34] as may retrospective recall over a reasonably long (12 months) period [29,35].

We found differences in non-fatal injury rates by geographical regions: low land and mid hill areas had higher rates when compared to the high hill and urban areas. Boys had higher rates than girls. Falls were the commonest cause of non-fatal injury to both the boys and girls; almost half of all injuries occurred in home environments. The rates were higher among the children of age groups 1–4 years and 5–9 years. This is not borne out by our findings in the lower altitudes of Nepal but intriguingly supported for the higher altitude dwellers.

### 4.1. Strengths 

This study covered a variety of geographical sub-regions within the Makwanpur district: Mountains (high hill), middle hills and plains (low land) areas. The use of an existing list of households from which to draw sample households was the biggest technical strength of this study which is not usually available in low-income countries. Standard, validated survey tools recommended by the WHO were used after appropriate modifications. Local and experienced interviewers carried out the survey. As in other developing countries [36,37], the response rate was very high.

### 4.2. Limitations

The information about child morbidity relied on proxy responses: Parents, preferably mothers, were considered the most appropriate person to be interviewed if available. Proxy respondents may not always know what children were doing at the time of injuries. Studies have found very high discrepancy in reported child morbidity rates using proxy respondents other than mothers [38]. Responses may vary with parents’ awareness of injury and other health issues which also may relate to parents’ education and occupation.

This study used a recall period of one year for non-fatal injuries which was useful to cover seasonality of injuries [38] but this may be too long for people to remember details of injuries [39]. A separate qualitative study conducted in Makwanpur district alongside this study suggested that injuries may be ignored or not recognised by the community and a one year recall period might have resulted in some underreporting [5]. This study included all injuries therefore people might have remembered some and forgotten others.

During the study, we asked households if any child had died from an injury during the previous 5 years. Ten fatal injuries were reported, and no further analysis is reported here due to the small numbers. The recall period for fatal injuries was set for 5 years because it was assumed that people would remember fatal cases for a much longer time. The survey relied on responses of the family member about the number of deaths as death registration was not widely practiced in Nepal. The sample size for the survey was estimated using data for non-fatal injuries, not fatal ones. Therefore a larger survey would have been required to have reported the epidemiology of fatal injuries. However fatal child injury was not the focus of this paper.

Issues about translation and understanding of terms and definitions of injuries need to be considered. There were really interesting challenges that arose during the study regarding understanding of injuries being preventable and not due to fate [5], and the challenge of reporting “accidents” when that term is not easily translated into Nepali. A qualitative study [40] done as part of this research revealed community perspectives on occurrence, factors influencing childhood injuries, their treatment and prevention practices in the communities; and also explored the existing infrastructure of keeping children safe from injuries. Poisoning, near-drowning and injuries occurred while working on farms *etc.*, might have been ignored in remembering something more severe. Beattie and colleagues have discussed the issues around scoring of severity of non-fatal injuries in children [41].

## 5. Conclusions 

This study is unique in the context of Nepal where considerable demographic, social and political changes are underway. In this context, this study highlights the current burden of injuries in the child population of Nepal and the need for focused activities for child injury prevention. Such activities include identification of effective injury prevention strategies, preparation of injury prevention policies and implementation of injury prevention activities. Interventions aimed to increase awareness about injuries, their risk factors and their prevention at community level could be helpful to prevent children from premature deaths and disability. Interventions which promote child supervision can be tailored to local community needs to minimise injury risk. It may be confidently predicted that the rise in the number of vehicles in Nepal will result in an increasing burden of death and disability resulting from traffic related injury in the absence of effective interventions. Children’s exposure to risks and hazardous environments has been exacerbated by recent major earthquakes in Nepal; interestingly injury prevention is not yet an agenda [42]. Future reconstruction and rebuilding activities will continue for some years, further exposing children to hazards.
